# The Central Role of Biometals Maintains Oxidative Balance in the Context of Metabolic and Neurodegenerative Disorders

**DOI:** 10.1155/2017/8210734

**Published:** 2017-07-02

**Authors:** Michal Pokusa, Alžbeta Kráľová Trančíková

**Affiliations:** ^1^Jessenius Faculty of Medicine in Martin, Biomedical Center Martin JFM CU, Comenius University in Bratislava, Bratislava, Slovakia; ^2^Department of Pathophysiology, Jessenius Faculty of Medicine in Martin, Comenius University in Bratislava, Bratislava, Slovakia

## Abstract

Traditionally, oxidative stress as a biological aspect is defined as an imbalance between the free radical generation and antioxidant capacity of living systems. The intracellular imbalance of ions, disturbance in membrane dynamics, hypoxic conditions, and dysregulation of gene expression are all molecular pathogenic mechanisms closely associated with oxidative stress and underpin systemic changes in the body. These also include aspects such as chronic immune system activation, the impairment of cellular structure renewal, and alterations in the character of the endocrine secretion of diverse tissues. All of these mentioned features are crucial for the correct function of the various tissue types in the body. In the present review, we summarize current knowledge about the common roots of metabolic and neurodegenerative disorders induced by oxidative stress. We discuss these common roots with regard to the way that (1) the respective metal ions are involved in the maintenance of oxidative balance and (2) the metabolic and signaling disturbances of the most important biometals, such as Mg^2+^, Zn^2+^, Se^2+^, Fe^2+^, or Cu^2+^, can be considered as the central connection point between the pathogenesis of both types of disorders and oxidative stress.

## 1. Introduction

### 1.1. Contemporary Concept of Oxidative Stress

The traditional concept of oxidative stress is based on an imbalance between the production of free radicals, namely, reactive oxygen species (ROS) and reactive nitrogen species (RNS), and the antioxidant capacity of the organism. The normal function and survival of eukaryotic organisms is fully dependent on oxygen and energy metabolism. Differences in the oxygen demands of the various tissues follow their special metabolic requirements. Oxidative damage is elevated in proportion to higher oxygen consumption under diverse pathological metabolic conditions [1]. Damaging effects of this phenomenon can be observed in intracellular metabolism and also in the structural features of cells [2]. Free radicals, the active compounds in oxidative damage, are defined as molecules with unpaired electrons in their outermost orbit. A typical oxidizing substance involved in the production of free radicals in living systems is oxygen [3].

Mitochondria together with several other eukaryotic cellular compartments such as plasma membrane [4, 5], cytosol [6, 7], peroxisomes, lysosomes [8], and endoplasmic reticulum (ER) [9, 10] significantly participate in ROS production and its consequent utilization [11]. In mitochondria during aerobic metabolism, the reduction of excessive electronegative oxygen atoms leads to the formation of reactive intermediates such as superoxide that can easily be converted to various forms of ROS. These include the superoxide anion radical, hydroxyl radicals, and the nonradical hydrogen peroxide. In this process, complex I (NADH-ubiquinone oxidoreductase) and complex III (ubiquinol-cytochrome c oxidoreductase) of the respiratory chain are the two main locations of ROS production [12, 13]. In addition, the ER, because of the activity of cytochrome P450-dependent oxygenases, [9, 10] and cytosolic xanthine oxidase provide another source of ROS [6, 7]. Peroxisomes show a very interesting example of high ROS production. They play a crucial role in many metabolic processes including fatty acid oxidation, metabolism of amino acids, and synthesis of lipid compounds [14], and most enzymes catalyzing these processes produce ROS during their activity [15]. Several studies indicate that a high proportion of peroxide (20–60%) is generated inside peroxisomes and diffuses through the peroxisomal membrane protein 2 (Pxmp2) channel to the surrounding medium [16]. Moreover, in 1972, Boveris and colleagues showed that, in rat liver, the major proportion of peroxide (about 35%) is generated by peroxisomal oxidases [17].

ROS, especially superoxide anion, can be generated nonenzymatically, when the oxygen directly accepts a single electron by the reduced coenzymes, prosthetic groups (e.g., flavins or iron-sulfur clusters), or previously reduced xenobiotic [13, 18].

Other reactive species, such as RNS, are also produced in eukaryotic organisms by cell metabolism [19]. Nitric oxide (NO) is generated by the cytosolic nitric oxide synthases (NOS), which convert L-arginine into L-citrulline and NO [20, 21]. To date, three different isoforms of NOS have been identified, depending on their cellular localization and cellular function. The activity of neuronal NOS (nNOS) and endothelial NOS (eNOS) is regulated by transient interaction with Ca^2+^/calmodulin [18, 22]. Inducible NOS (iNOS) is not regulated by Ca^2+^, but its activity is induced by infection, inflammation, or trauma [18, 22].

Both ROS and RNS play dual roles in cell metabolism. On one hand, at the physiological level, both ROS and RNS play important and beneficial roles in various cellular processes. For example, ROS are involved in growth, apoptosis, and gene transcription, and at the system level, they support complex functions, such as the regulation of blood pressure, cognitive function, or immune response [6, 23]. RNS contribute to the regulation of apoptotic and necrotic cell death [6], and at the systemic level, RNS also contribute to blood vessel modulation [24], proliferation, and relaxation of vascular smooth muscle cells, leukocyte adhesion, angiogenesis, and thrombosis [25]. On the other hand, their overproduction in cells and the resulting accumulation of oxidative damage lead to lipid peroxidation, oxidative modification of structural proteins, protein misfolding and aggregation, and DNA mutation as a result of RNA/DNA oxidation [26] and additionally to chronic diseases such as neurodegeneration, cancer, diabetes, cardiovascular disease, stroke, and chronic inflammation [27, 28].

Thus, the cellular concentration of ROS and RNS clearly determines the alteration between their beneficial and harmful effects. However, the exact concentration of specific ROS and RNS at which this shift in function occurs remains unknown. Several authors have suggested that this phenomenon depends on the particular cell type, cellular compartment, time, source of their production, and, of course, the type of ROS and RNS generated [6, 29].

Tissue defense against oxidative damage is based on the antioxidant capacity of exogenous antioxidant molecules such as ascorbate and vitamin E. In addition, endogenic molecules, such as glutathione (GSH), catalase, and the superoxide dismutases (SOD), provide the main antioxidant capacity of living eukaryotic cells. In general, a tight relationship exists between the activity levels of these enzymes and the concentration of various biometals, usually serving as cofactors of these enzymes. For example, copper (Cu^2+^) and zinc (Zn^2+^) ions, in particular, have a great impact on the activity of cytoplasmic SOD, whereas manganese (Mn^2+^) is a metal essential to the function of the mitochondrial type of this enzyme (mSOD) [30, 31]. Iron ions (Fe^3+^) are an integral component of catalase [32]. Almost no antioxidant enzymatic action can be managed without a specific ion equilibrium. Aberrations in the plasma concentrations of magnesium (Mg^2+^), Cu^2+^, Zn^2+^, and selenium (Se^2+^) ions are observed together with oxidative stress markers in clinical studies of metabolic or neurodegenerative disorders [33, 34].

When the antioxidant capacity of these molecular instruments is insufficient to bring the free radicals back to the basic nonreactive state, organic molecules such as DNA, RNA, lipids, and enzymes are the main targets of oxidative events mediated by the ROS [35]. The intracellular machinery, which secures redox balance, is similar to other cellular systems and is able to adapt. Signaling pathways, which regulate the strength of antioxidant capacity, act mainly through the activation of the antioxidant response element (ARE) in the promoter regions of genes by means of NF-E2-related factor 2 (Nrf2). The binding of ROS-sensing basic leucine zipper (bZIP) transcription factor Nrf2 to the promoter ARE region (Nrf2/ARE) results in the upregulation of the expression of a wide range of antioxidant enzymes [36]. In addition to ROS or RNS, prolonged hyperglycemia (increased level of sugar) with proinflammatory advanced glycation end (AGE) products can also lead to the activation of the Nrf2/ARE signaling pathway [37].

Because events involved in the maintenance of the oxidative balance can be observed in most cell types in the body, the defects in these processes are of major importance in the development of systemic changes in the inflammatory response, energy metabolism, membrane dynamics, or tissue regeneration. All these activities are the basis for the pathogenesis of all types of metabolic and neurodegenerative disorder. The main objective of this review is to focus on the common features shared by these two distinctly different disorders in the way that the cellular oxygen balance is preserved. Emphasis is placed on the central physiological role of metal ions relevant to pathogenesis of both types of disorders. The assessment of available information concerning the oxidative background of both metabolic and neurodegenerative disorders might contribute to the identification of oxidative stress as one of the main causes responsible for the metabolic roots of neurodegenerative disorders.

## 2. Oxidative Stress and Metabolic Disorders

**Figure 1:**
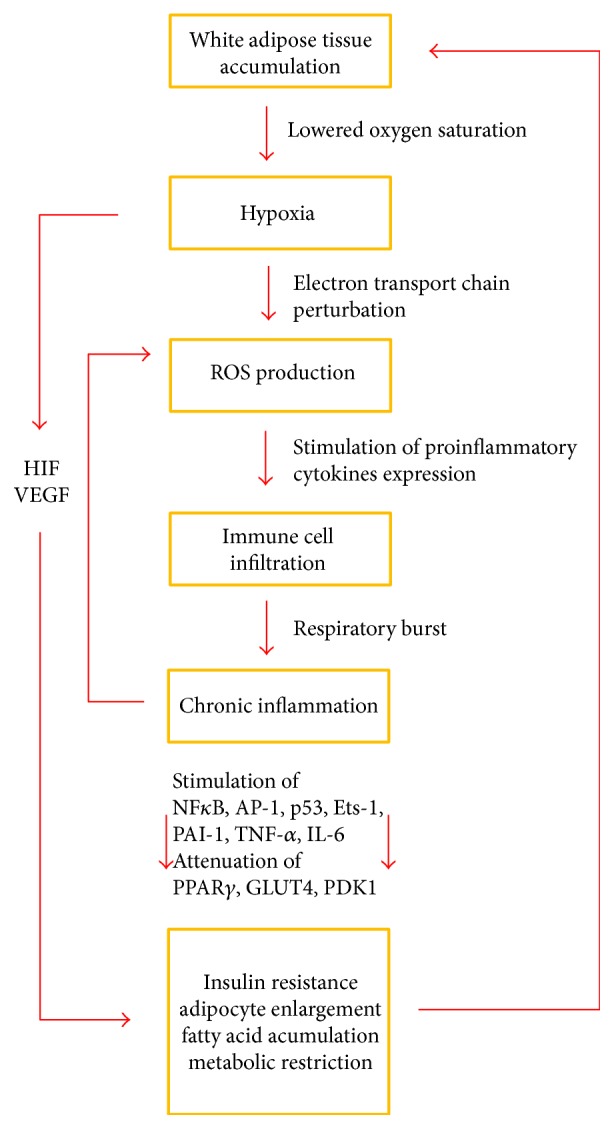
The progression of pathological changes in adipose tissue after initial fat accumulation with a focus on participation of hypoxic condition. Lowered oxygen saturation in enlarged adipose tissue leads to hypoxic conditions. Mitochondrial dysfunction in hypoxic tissue causes alterations in the electron transport chain and thus an increase in generated ROS, which are critical for the further activation of immune cells and the development of chronic inflammation. The activation of relevant genes leads to the pathogenesis of metabolic disorders and creates the vicious cycles further empowering the pathological changes. The interconnections between the pathological processes leading to the final metabolic disorders are marked in boxes. Red arrows indicate the flow of changes and the vicious cycles between each pathological stage, together with the causative effect and the ablation of gene expression between specific grades of pathogenesis.

Randomly produced ROS not only have harmful effects but also exhibit a physiological role in the innate immune response after the respiratory burst of immune cells. Several chronic diseases, including metabolic disorders, may be partly caused by the constant activation of the immune system, which might further result in tissue and also systemic oxidative stress. The reduction of oxygen to hydrogen peroxide or peroxynitrite, which takes place in immune cells, for example, neutrophils during the respiratory burst, is based on the activity of NADPH oxidase (NOX) and iNOS [38, 39]. Higher local expression of ROS-producing NOX is tightly bound to the elevated levels of hypoxia-inducible factor 1 (HIF-1) and factors responsible for angiogenesis (vascular endothelial growth factor (VEGF)). These proteins are the key players in processes improving tissue oxygenation ability during hypoxic or other pathological conditions that lead to an energy deficit attributable to substrate oxidation [40]. Oxidation-sensing factor, HIF-1*α*, and VEGF are often discussed in the context of obesity, because cellular hypoxia has been observed in the adipose tissue of obese individuals [41]. During hypoxia, ROS production is rapidly elevated. The precise mechanisms are still not known, but oxygen deprivation and its impact on the mitochondrial electron transport chain have to be taken into consideration. ROS and also RNS themselves are similarly responsible for the activation of broad ranges of proinflammatory factors such as nuclear factor-*κ*B (nuclear factor *κ*-light-chain-enhancer of activated B cells; NF-*κ*B), activator protein-1 (transcription factor; AP-1), cellular tumor antigen p53, and protein C-est-1 (ETS proto-oncogene 1, Ets-1; transcription factor) together with proliferatory and hypoxia sensing factors VEGF and HIF [42]. Moreover, proinflammatory NF-*κ*B itself can stimulate HIF-1*α* basal expression by binding to the HIF gene promotor region [43, 44], possibly serving as an explanation of this inflammation and hypoxia-sensing switching point. As is summarized in the scheme in Figure 1, the upregulation of HIF-1*α* in hypoxic adipose tissue under obesity conditions is, however, positively correlated with the exacerbation of insulin resistance and glucose intolerance [45]. This negative effect of HIF-1*α* on glucose tolerance is mediated by the attenuation of adipogenic factors such as peroxisome proliferator-activated receptor *γ* (PPAR*γ*), glucose transporter type 4 (GLUT4), and pyruvate dehydrogenase lipoamide kinase isozyme 1 (PDK1) and is associated with the metabolic deprivation of adipocytes together with fatty acid accumulation [46]. The disruption of HIF-1*α* in adipocytes of a transgenic mouse model has been shown to improve the metabolic function of these adipocytes and to ameliorate insulin resistance [47].

All of this evidence strengthens ideas regarding the cooperative effects of hypoxia and inflammation in the pathophysiology of metabolic disorders. However, a rough collection of data obtained in recent years indicates that proinflammatory cytokines secreted by immune-competent cells and adipocytes might in turn trigger the development of insulin resistance. On the other hand, anti-inflammatory medication may reverse this process [37]. The infiltration of macrophages into adipose tissue is tightly bound with an excessive accumulation of fat in obesity and with the secretion of proinflammatory cytokines, such as plasminogen activator inhibitor-1 (PAI-1), tumor necrosis factor *α* (TNF-*α*), and interleukin6 (IL-6). Proinflammatory cytokines can also originate in white adipocytes, which have self-endocrine potential. In white adipose tissue, such an enhanced secretory capacity for proinflammatory cytokines creates an even stronger background for the development of low-grade inflammation and related oxidative stress [48].

Adipokines, which are endocrine-active substances originating from white adipocytes, are critical factors contributing to the regulation of free radical formation. Leptin, an adipokine, whose secretion capacity is dependent on total adipose tissue mass, is well known for its restrictive effect on food intake. Because of these anorexic effects, leptin has been considered as a potential therapeutic agent for obesity. Contrary to its beneficial effects, a higher leptin concentration in blood (hyperleptinemia) is well known to elevate the level of oxidative stress by the stimulation of mitochondrial and peroxisomal oxidation of fatty acids. Stimulation of fatty acid utilization can be understood as beneficial in obesity, but the pro-oxidative stimulation of mitochondrial and peroxisomal metabolism is a critical factor in ROS generation. An increase in mitochondrial metabolism during hyperleptinemia is also observed in immune cells. This effect has been suggested to be related to the proliferation and activation of monocytes infiltrating the adipose tissue [49]. Leptin, by promoting pro-oxidative events, increases the phagocytic activity of macrophages and also induces the synthesis of proinflammatory substances such as IL-6 and C-reactive protein (CRP) [50]. In addition, adiponectin, another adipokine secreted by adipocytes, shifts macrophages towards the anti-inflammatory phenotype. However, proinflammatory cytokines such as TNF-*α* and IL-6 inhibit its synthesis [51]. This suggests that, in contrast to leptin, adiponectin acts as an anti-inflammatory agent. Study of the 3T3-L1 adipose cell line has shown that one of the stimulatory effects of adiponectin secretion is mediated by both insulin and amino acids [52]. Another study of animal models has proposed that adiponectin allows insulin action by its stimulatory effects on glucose uptake through the activation of the AMP-activated protein kinase (AMPK) [53]. In agreement with these observations, low adiponectin levels in plasma are associated with insulin resistance, as has been seen in obese patients [54]. On the other hand, insulin resistance is positively correlated with the blood level of a third type of adipokine called resistin, which is similar to leptin being associated with the stimulation of the secretion of proinflammatory molecules [55, 56]. The physiological effects of resistin have been suggested to be associated with glucose storage, as it has been observed to be elevated during long-term physical exercise [57]. Moreover, the ablation of the resistin gene in mice stimulates the regulation of gluconeogenic enzymes [58]. Low-grade inflammation of excessive white adipose tissue mass may therefore result in the chronic pathologic upregulation of resistin plasma levels, accompanied with the development of glucose intolerance. In agreement with these data, insulin resistance and hyperglycemia in a rat metabolic toxicity model treated with hyperglycemia-causing agent hydrochlorothiazide (HCTZ) and a high-fat diet have been associated with higher levels of malondialdehyde (oxidative stress marker, ROS-induced metabolite of polyunsaturated lipids) [59].

Insulin resistance, as the main pathogenic factor of type II diabetes mellitus (T2DM), has a dramatic impact on energy substrate distribution, accompanied by the modification of mitochondrial function. A study by Anderson and colleagues has clearly shown that the mitochondria of obese and insulin-resistant rodents and humans produce elevated levels of ROS when compared with those of their lean counterparts [60]. A higher intake of energy substrates by obese individuals enhances the proton gradient of the inner mitochondrial membrane with a higher probability of electron leakage from the terminal respiratory chain. This uncontrolled disruption of electron potential is usually associated with ROS generation [61]. In cases of developed insulin resistance, a lower glucose intake, and the starving of the cells, ROS has a negative effect on the ATP concentration and thus leads to the upregulation of the burning of fatty acids by beta-oxidation. The ATP production by *β*-oxidation is associated with the same effect on transmembrane potential magnification. Whether the enhanced generation of ROS is the cause of insulin sensitivity impairment or vice versa, the effect of higher nutrient intake is still not fully understood [62]. The effectiveness of antioxidant therapy in obesity and T2DM is the subject of intensive discussion [62, 63].

Altered mitochondrial function, which is behind the onset of insulin resistance and the onset of metabolic syndrome, might be based on mitochondrial dysfunction related to mitochondrial cytopathies [64]. The term “mitochondrial dysfunction” can be considered as general pathophysiological alterations that result in diminished antioxidant defense through ROS production, reduced oxidative phosphorylation, and decreased ATP production [65]. A decreased level of the elimination of damaged mitochondria may be the result of alterations in mitochondrial fission and fusion processes and the inhibition of mitophagy [66]. On the contrary, the stimulation of mitochondrial biogenesis improves metabolic status and is considered to be protective against the development of T2DM. Genes, such as PPAR*γ*, peroxisome proliferator-activated receptor *γ* coactivator 1 (transcriptional cofactor; PGC1-*α*), nuclear respiratory factors 1 and 2 (NRF1 and 2), and mitochondrial uncoupling protein 1 (UCP1), which are involved in the regulation of mitochondrial biogenesis, are upregulated not only by the stimuli of physical activity and myogenesis but also by dietary restriction and low temperature [67]. Quantitative real-time polymerase chain reaction (PCR) carried out in the 3T3-L1 adipose cell line has revealed that the supplementation of growth medium by balanced deep-sea water with higher concentrations of various ions such as Ca^2+^ and Mg^2+^ leads to an increase in the expression of genes involved in mitochondrial biogenesis in preadipocytes, such as PGC1-*α*, NRF1, and mitochondrial transcription factor A (TFAM) [68].

## 3. Biometals in Metabolic Disorders

The precise role of biometals in metabolic modulation has not been fully uncovered, but the available data support the hypothesis of a strong relationship between these trace elements and essential hypertension, endothelial dysfunction, insulin resistance, oxidative stress, and the atheroinflammatory state. The pathological activation of the immune system might be a consequence of disturbed ion homeostasis. The activation of immune cells leads to an intracellular increase of their Ca^2+^ concentration as a potential consequence of ion misbalance, especially that of biometals [69]. As summarized in Table 1, numerous clinical studies emphasize the increase/depletion or disturbances of biometal ratios in the pathophysiology of metabolic disorders [33, 70, 71]. Bioactive metals such as Mg^2+^, Zn^2+^, Se^2+^, Cu^2+^, and Mn^2+^ are collectively considered as antioxidant trace elements [70, 72]. They act as cofactors for antioxidant metalloenzymes [72, 73]: Cu^2+^ and Zn^2+^ have been identified as cofactors of cytoplasmic superoxide dismutase (Cu-Zn-SOD) [31] and Mn^2+^ as a cofactor of mitochondrial SOD (Mn-SOD) [30].

### 3.1. Magnesium

In general, Mg^2+^ has one of the most important roles in the regulation of metabolism. A negative correlation of insulin resistance and hyperglycemia with Mg^2+^ plasma concentration has been observed in rats after their administration with the hyperglycemia-causing agent HCTZ [59]. Obese subjects frequently exhibit metabolic disorders together with elevated inflammatory markers, such as the C-reactive protein (CRP) and alanine transferase (ALT), markers associated with an increased risk of cardiovascular disease and liver damage. Several studies have described the depletion of magnesium concentrations in the blood of obese individuals [74, 75], and this effect seems to be correlated with a greater degree of oxidative stress [76]. Decreased Mg^2+^ levels, together with an increased concentration of malondialdehyde (an oxidative stress marker), have been found in blood samples, in particular, in younger insulin-resistant patients in comparison with healthy controls [77]. Moreover, the status of glucose and triglyceride metabolism has been significantly improved in magnesium-supplemented pregnant women suffering from gestational diabetes mellitus [78]. Oral supplementation by magnesium chloride to obese women reduces plasma ALT levels, together with a tendency towards a reduction in CRP levels [79]. Furthermore, magnesium as a cofactor plays a role in glutathione (GSH) production by gamma-glutamyl transpeptidase [80]. Therapeutical treatment by magnesium sulphate in another study has been shown to lead to the increased activity of superoxide dismutase and catalase [81].

As a cofactor of pyruvate dehydrogenase phosphatase (PDP), Mg^2+^ also facilitates the dephosphorylation of pyruvate dehydrogenase (PDH) and, thus, its activation. PDH is the rate-limiting enzyme guiding the intermediate metabolites from anaerobic glucose breakdown to oxidative metabolic pathways [82]. Because of these specific roles, Mg^2+^ can speculatively be considered as an ion of the aerobic/anaerobic switch of glucose degradation. Furthermore, according to observations of Kelley and colleagues, PDH activity is below the physiological level in the skeletal muscle of patients with T2DM [83]. From another point of view, diabetic patients are well known to have problems with the maintenance of ion homeostasis, because of the high prevalence of nephropathy within these patients. Hypothetically, this mechanism might be a link between a lower Mg^2+^ concentration and a lower retention capacity of DM kidneys, particularly in the case of nephropathic comorbidity [84]. Under healthy conditions, insulin activates the reuptake of Mg^2+^ by the activation of transient receptor potential melastatin type 6 (TRPM6) channel [85]. However, in the absence of insulin signaling, such as during insulin resistance, Mg^2+^ reuptake may be also impaired. Even more interesting is the observation that oxidative stress reduces TRPM6 activity [86].

**Figure 2:**
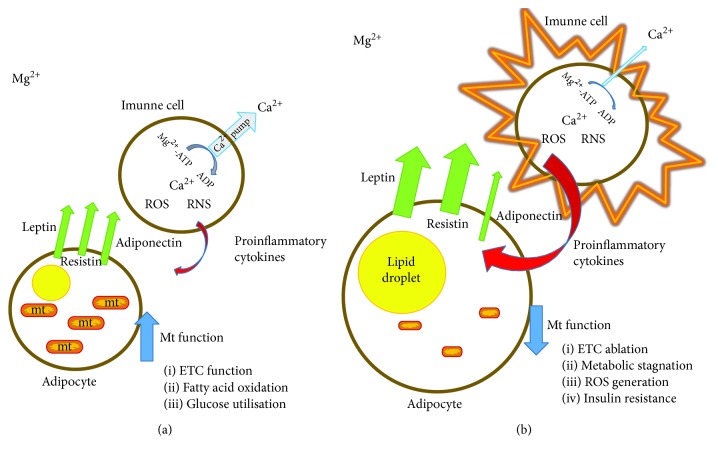
Schematic illustration demonstrating the relationship between adipocytes and immune cells in white adipose tissue with an accent on magnesium regulatory functions. (a) Normal magnesium levels preserve the standard physiological activity of the ATP-dependent Ca^2+^ pump. A low intracellular concentration of calcium keeps immune cells in an inactive state. (b) A decrease in the magnesium concentration reduces the excretion rate of calcium ions from cells via the Mg^2+^-dependent Ca^2+^ pump. Activated immune cells in adipose tissue secrete proinflammatory cytokines with inhibitory effects on adipocyte metabolism. Genes responsible for mitochondrial biogenesis (PPAR*γ*, PGC1-*α*, NRF1–2, and UCP) are attenuated under proinflammatory conditions, leading to the attenuation of mitochondrial functions. Higher expression of leptin is connected to an increase in fat tissue mass caused by metabolic stagnation. Upregulation of resistin is complementary to the stimulated expression of proinflammatory cytokines. Adiponectin secretion is attenuated by proinflammatory cytokines resulting in the aggravation of glucose tolerance and the development of insulin resistance.

The inhibition of the erythrocyte ions of the Na^+^/K^+^ and Ca^2+^ ATPases in hibernated black bears is further evidence of ion channel regulation by oxidative stress or hypomagnesemia [87]. During hibernation, these bears suffer from a higher degree of oxidative stress, since a higher level of oxidative stress marker malondialdehyde has been detected in their blood. Actually, the activity of both these ion pumps is regulated by Mg^2+^-dependent phosphorylation. In general, the presence of Mg^2+^ or of some other divalent cations (Mn^2+^, Co^2+^, and Fe^2+^) is essential for ATP hydrolysis at significant rates and is crucial for the action of all ATPases, including the Ca^2+^ pump. An explanation for this effect of Mg^2+^ is its participation in the formation of a complex with ATP, thereby facilitating the hydrolysis of ATP to fuel the Ca^2+^ pump (Figure 2). By this mechanism, Mg^2+^ represents the natural antagonist of calcium effects in living systems, by mediating the excretion of this ion through the cytoplasmic membrane [54, 88].

The concentration of Ca^2+^ should be tightly regulated under all conditions, mainly in the regulation of the immune response. A strong association of the hyperactivity of immune cells, Ca^2+^ concentration, and Mg^2+^ deficiency can be found in the literature [89, 90]. Rats fed on an Mg-deficient diet for eight days show a significant increase in intracellular Ca^2+^ concentrations after the administration of platelet-activating factor, compared with controls [91]. According to another group of authors, a short-term deficiency of Mg^2+^ (21 days) in rats leads to an increase of a broad variety of cytokines such as IL-1*α*, IL-1*β*, IL-2, IL-6, and TNF-*α* [92]. Furthermore, a study performed by Bussiere and colleagues has demonstrated the upregulation of a wide variety of genes associated with the immune response of neutrophils in Mg-deficient rats compared with that in control rats. The authors have also identified genes involved in apoptosis, coding heat shock proteins, cytoskeletal proteins, and proteins implicated as stress response regulators and effectors and enzymes implicated in thromboxane synthesis. These genes have been named by the authors as a genes implicated in the immunoinflammatory process of Mg^2+^ deficiency [93]. On the contrary, studies focused on a higher magnesium concentration as a result of its addition to growth media have identified several significant effects of the elevated concentration of this biometal in immune cells. In isolated human leukocytes, magnesium-supplemented growth media lead to a decrease in the intracellular Ca^2+^ concentration and, furthermore, to a significant decrease in immune response activation by chemotactic factor FMLP (N-formylmethionyl-leucyl-phenylalanine) [94]. In addition, leukocyte activity reduction, in response to magnesium supplementation in growth medium followed by Ca^2+^ reduction in cell models, can be explained by the regulatory mechanism of Mg^2+^ on Ca^2+^ ATPase [95].

### 3.2. Zinc and Other Biometals in Metabolic Disorders

Metabolic disorders, such as essential hypertension and T2DM, are also associated with disturbances of other metal ion concentrations. Zinc, among the other biometals, is the second most prevalent trace element in the human body. In contrast to iron, zinc is a redox-inactive biometal and serves as an important component of more than 2700 enzymes including hydrolases, transferases, oxidoreductases, ligases, isomerases, and lysates [96]. This large number of enzymatic activities modulated by zinc explains the requirement for zinc in DNA, RNA, protein, and lipid synthesis, possibly explaining its major role in the preservation of the stability of the genome, proteome, and other biomolecules [97]. This action includes, but is not limited to, the antioxidant effects of zinc and its participation in DNA repair, the DNA damage response, and the synthesis of molecules (e.g., methionine) necessary for DNA methylation. Furthermore, it contributes to the maintenance of the redox equilibrium through various mechanisms. For example, zinc is an inhibitor of nicotinamide adenine dinucleotide phosphate (NADPH) oxidase, a cofactor of SOD, and induces the generation of metallothionein, a scavenger of oxidants [98].

Moreover, zinc is crucial for the normal development and function of cell-mediated immunity associated with T cells. The deficiency of this biometal also negatively influences the secretion of interleukin 1beta (IL-1*β*) by macrophages [99]. The altered production of cytokines during zinc deficiency can lead to inflammation as evidenced by the induction of IL-1*β* secretion in zinc-depleted macrophages [100]. In rats, a zinc-depleted diet for 4 weeks causes a significant increase in Mg^2+^ and Fe^2+^ concentration in serum. In particular, the elevation of iron ions in the extracellular space is associated with oxidative stress induction [101]. According to Gouaref and colleagues, serum concentrations of Zn^2+^ are significantly decreased in patients suffering from essential hypertension and T2DM. Several other types of metabolic disorders are, unlike the lowering of zinc, accompanied by higher levels of Cu^2+^ [33]. Both Zn^2+^ deficiency and an excess of Cu^2+^ have been associated with an increased risk of T2DM and cardiovascular diseases. In addition, in hypertensive patients, but not in T2DM patients, the Zn^2+^/Cu^2+^ ratio is significantly decreased. This suggests that the Zn^2+^/Cu^2+^ ratio reflects the transition from the hypertension phase to T2DM-associated hypertension [33]. In agreement with these observations, a higher Zn^2+^/Cu^2+^ ratio has been associated with a reduced risk of poor glycemic control in T2DM patients [102]. Oral administration of zinc together with acetylsalicylic acid to T2DM rats reduces plasma glucose levels and prevents diabetic cardiomyopathy [103]. Positive effects of zinc supplementation have been also investigated in a study of obese mice fed on a high-fat diet (HFD) [104]. HFD significantly decreases the expression of transcription factor Nrf2 in the *aorta tunica media* in a time-dependent manner. Zinc deficiency aggravates the downregulation of this transcription factor, which is associated with the stimulation of antioxidant genes through the ARE in the promoter regions of the relevant genes, as mentioned above [36]. Zn^2+^ supplementation prevents the decrease in aortic *Nrf2* expression induced by HFD [104].

Several authors have also investigated the association of the homeostasis of other ions with metabolic disorders. Yadav and colleagues have observed, in addition to reduced levels of Zn^2+^ and Mg^2+^, a reduction in Se^2+^ serum levels in insulin-resistant individuals compared with those in healthy controls [105]. Serum concentrations of Se^2+^ together with Zn^2+^ are, according to Gouaref and colleagues, significantly decreased in patients suffering from essential hypertension and T2DM. On the contrary, several types of metabolic disorders are accompanied with higher levels of Cu^2+^ and Mn^2+^ [33].

## 4. The Role of Oxidative Stress in Neurodegenerative Disorders

The higher susceptibility of the brain to oxidative stress arises from its extraordinary utilization of oxygen. Despite the brain sharing only 2% of body mass, it consumes approximately 20% of the total oxygen production [106]. In particular, neurons and astrocytes, which are the two major cell types in the brain and whose function is fully dependent on oxygen and glucose, consume approximately 10-fold more oxygen compared with other cells [107]. Moreover, neurons are nondividing cells with a long life duration; therefore, they are heavily exposed to the accumulation of oxidative stress. In addition, redox-active metals, which play an active role in ROS production, are abundant in the brain [108]. Despite this fact, neurons do not possess an extra antioxidant capacity or special antioxidant systems.

**Figure 3:**
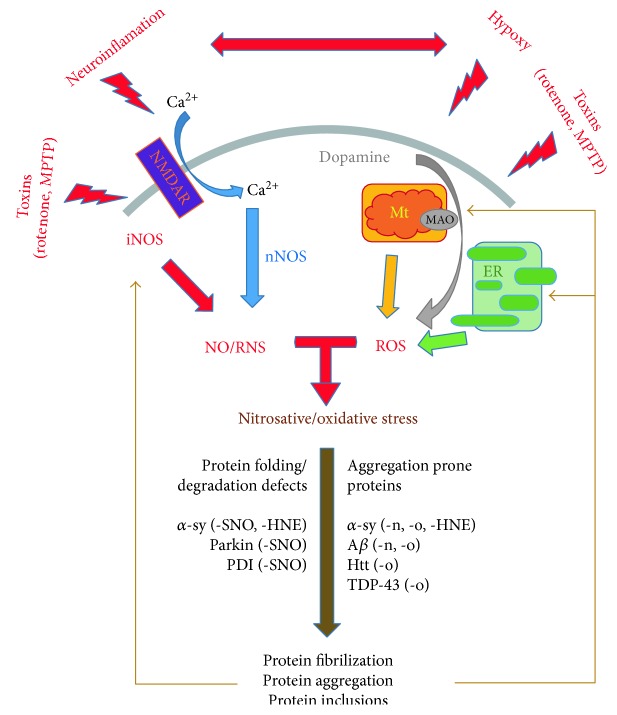
The causative mechanisms of the pathological elevation of nitrosative/oxidative species and their abilities to modify relevant proteins associated with the pathogenesis of neurodegenerative diseases. The activation of iNOS by inflammatory processes and of nNOS by Ca^2+^ influx through NMDAR leads to an increase in intracellular levels of RNS. On the other hand, ROS is generated upon mitochondrial dysfunction, and endoplasmic reticulum (ER) stress is a result of dopamine metabolism by monoamine oxidase (MAO). Hypoxic conditions and toxic compounds, such as rotenone and MPTP, are also considered as carriers of harmful effects to the physiology of the mentioned organelles. However, rotenone and MPTP also exert stimulatory effects on iNOS. Damaged and aggregated proteins create a solid base on which to create a vicious cycle by the strengthening of iNOS activity and by the deepening of mitochondrial and ER dysfunctioning.

In mammalian cells, RNS, physiological messenger molecules, are normally produced at very low levels. In neurons, NO and RNS are generated by Ca^2+^-activated nNOS and neuroinflammatory stimuli-activated iNOS [109, 110]. nNOS activity requires the triggering of N-methyl D-aspartate-type glutamate receptors (NMDAR), which promote Ca^2+^ influx into the cells (Figure 3). Furthermore, activated NMDAR also leads to the generation of ROS [111]. In terms of neurodegenerative diseases, amyloid *β* (A*β*) oligomers or 1-methyl-4-phanel-1,2,3,6-tetrahydropyridine (MPTP) leads to an increased NO production and neurotoxicity via the stimulation of iNOS expression (Figure 3). Moreover, iNOS knockdown or knockout protects cells against the MPTP-induced neurotoxicity in animal models [112].

The selective vulnerability of certain neuronal populations, which are affected in a progressive and irreversible manner, is a common feature of neurodegenerative diseases. These neuronal populations are usually the first that show functional degeneration and cell death during aging and, even more prominently, during neurodegenerative diseases [113]. Several decades ago, observations that decreased levels of GSH and increased levels of lipid peroxidation and protein oxidation are commonly present in the brain tissues of patients with Alzheimer's disease (AD) or Parkinson's disease (PD) patients suggested that ROS/RNS accumulation is involved as a major pathogenic process in age-related and neurodegenerative disorders [19, 28, 114–116]. Despite oxidative stress, which is a common pathological mechanism, the vulnerability of diverse neuronal populations to oxidative damage varies in the different neurodegenerative diseases and within the neuronal population in a certain brain region. For example, the *entorhinal cortex* and the hippocampus CA1 region are the most affected brain regions in AD patients, whereas dopaminergic neurons in the *substantia nigra* represent the most vulnerable neurons in PD brains. Interestingly, dopaminergic neurons in PD brains are affected, whereas the ventral tegmental area (VTA) neurons are not [7]. This phenomenon is most probably attributable to the different degrees of oxidative stress present in the different neuronal populations and to the different expression profiles of the antioxidant systems [7]. For example, hippocampal CA1 neurons, compared with CA3 neurons, express a higher level of ROS-producing genes and, thus, are exposed to a higher level of oxidative stress. In dopaminergic neurons, ROS are generated, on one hand, as a result of dopamine metabolism by monoamine oxidase (MAO) (Figure 3) but, on the other hand, as a result of dopamine auto-oxidation [7, 113].

Taking this into consideration, we have to postulate the existence of additional molecular factors that regulate the selective loss of certain neuronal populations. The “multiple-hit hypothesis”, one of the suppositions for the development and progression of neurodegenerative disease, involves the interplay between molecular pathways in a sequential order [117–121]. This means that the neurodegenerative process is a result of the combined toxic stress from dopamine oxidation or mitochondrial function impairment, together with the failure of neuroprotective mechanisms, including the loss of function of parkin, the failure of antioxidant pathways, or stress-induced autophagic degradation [120]. In the context of energy metabolism, one more aspect should be adequately considered. Oxygen or glucose insufficiency in sensitive brain regions is associated with the overexpression and lowered clearance of A*β* in AD [122, 123]. Hypoxic tissues with reduced mitochondrial function are far more vulnerable to ROS-induced oxidative damage [124].

### 4.1. Involvement of ROS/RNS in Protein Folding and Aggregation as a Hallmark of Neurodegenerative Diseases

Similarities in pathological mechanisms underlying neurodegenerative diseases result from aberrant protein folding, the consequent aggregation of disease-specific proteins in cells, and the presence of ubiquitinated inclusion bodies. The relationship between protein misfolding and aggregation and excessive ROS/RNS production is well documented, although the exact mechanism of this pathological process is not fully uncovered. For example, ubiquitin E3 ligase (parkin, PARK2) and ubiquitin C-terminal hydrolase 1 (UCH-L1, PARK5) are critical for protein degradation via the ubiquitin-proteasome system (UPS). Mutations in parkin and UCH-L1, which often lead to their functional impairment and, thus, to the impairment of UPS, are both linked to PD [125–127]. Protein levels of UCH-L1 have been found to be downregulated in idiopathic PD and AD brains [128]. Significantly elevated levels of S-nitrosylated parkin (SNO-parkin) have been observed in the postmortem analysis of sporadic PD brains and in PD animal models [129, 130]. Furthermore, the overproduction of NO, for example because of MPTP or rotenone exposure (Figure 3), results in S-nitrosylation and the further oxidation of these proteins [128, 129, 131]. Upon S-nitrosylation, the E3 ligase activity of parkin is transiently increased, followed by its inhibition. The initial increase in E3 ligase activity enhances the ubiquitination of target proteins, the phenomenon observed in Lewy bodies (LB). The consequent inhibition of parkin activity impairs its ubiquitination activity and thus impairs UPS and protein degradation [110]. In this context, parkin in concert with PINK1 kinase (PARK6, another familiar PD-related gene) plays a role in mitochondria quality control and the subsequent removal of damaged mitochondria. Upon activation, PINK1 phosphorylates and activates parkin resulting in the ubiquitination of proteins of the outer mitochondrial membrane and consequently promotes mitophagy. Similar to the effect of SNO-parkin on UPS, the initial increase in parkin activity promotes mitophagy, whereas further exposure to NO induces the attenuation of mitophagy [132]. Additionally, UCH-L1 undergoes oxidative modification by 4-hydroxy-2-nonenal (HNE) leading to its abnormal function. An abnormal interaction with components of chaperone-mediated autophagy-dependent degradation (Lamp2a, Hsc70, and Hsp90) results in accumulation of chaperone-mediated autophagy substrates, such as *α*-synuclein (*α*-syn, PARK1, and PD-related gene), in cells [133].

Protein disulphide isomerase (PDI), a cellular defense protein, with chaperone and isomerase activity, plays a role in protein-folding quality control. PDI is upregulated as a response to ER stress induced by misfolded and aggregated proteins [9, 134]. This chaperone and protective effect of PDI is attenuated upon S-nitrosylation (SNO-PDI). Consistent with this information, increased levels of SNO-PDI have been detected in the brains of patients with PD, AD, and amyotrophic lateral sclerosis (ALS) [135, 136] and in response to iNOS activation in animal models of ALS [137] or in response to mitochondrial toxins, such as rotenone and MPTP, in cellular models [110, 135]. This indicates that, during neurodegenerative processes, proteins related to protein degradation, protein folding, and folding quality control undergo aberrant oxidative or nitrosative modifications, which result in the attenuation of the physiological function of these proteins.

Oxidative/nitrosative modification strongly impacts the structural properties of proteins directly linked with certain neurological disease; this occurs because of the ability of these modified proteins to form fibrillar units and formation of ubiquitin-positive inclusions in cells. For example, A*β* undergoes oxidative and nitrosative modifications that have been demonstrated to induce the formation of A*β* protofibrils and fibrils from monomeric A*β*. Nitrated and oxidized forms of A*β* have also been found in AD senile plaques. These modifications of A*β* have been suggested initially to stabilize the formed A*β* dimers and thus to induce plaque formation [138–140]. Moreover, the modifications of microtubule-associated protein tau (a protein linked with AD pathology) by HNE facilitate hyperphosphorylation and the consequent aggregation of tau and the major conformational changes of this protein, leading to neurofibrillary tangle formation [141–143]. In in vitro models, oxidized fatty acids also have a strong impact on tau polymerization. Transgenic animal models deficient in SOD2 or folate (folic acid, antioxidant) develop oxidative stress followed by tau phosphorylation and aggregation and the formation of amyloid A*β* plagues [144].

The misfolding and aggregation of *α*-syn represent the basic mechanism of dopaminergic neuronal loss associated with PD. Oxidative and nitrosative posttranslational modifications, including oxidation (o-*α*-syn), nitration (n-*α*-syn), and HNE modification (HNE-*α*-syn), facilitate the generation of protofibrillar structures and the further oligomerization of *α*-syn, with the highest impact of HNE-*α*-syn [145]. In in vitro studies of dopaminergic Lund human mesencephalic (LUHMES) neurons, HNE modification enhances *α*-syn interactions with membranes. HNE-*α*-syn exposure of differentiated LUHMES neuronal cells initiates intracellular ROS production followed by neuronal death. This can be effectively prevented by treatment with antioxidants [145].

As for previously discussed proteins, huntingtin (Htt; protein related to Huntington's disease (HD)) and TAR DNA-binding protein (TDP-43; protein related to amyotrophic lateral sclerosis (ALS)) undergo oxidative modifications with a similar effect on their conformational changes and protein aggregation [18].

## 5. Biometals in Neurodegenerative Disorders

The impaired cellular homeostasis of metal ions might initiate neurodegeneration through various mechanisms that have complementary roles in the pathogenesis of the different types of neural degeneration. These pathomechanisms include well-established oxidative stress, which is tightly bound to the incorrect generation of metalloproteins, the activation of microglial cells, and inflammation [146].

### 5.1. Iron

Iron is an important cofactor of many proteins, with a high redox flexibility, and plays a critical role in processes such as respiration, oxygen transport, nitrogen fixation, DNA synthesis and repair, and neurotransmitter synthesis [147–150]. Redox-active iron is directly linked with an increase in the generation of oxidative stress, together with inhibition of GSH activity with changes in the intracellular reduction potential attributable to GSH oxidation [150, 151]. Chelated reduced forms of iron do not participate in oxidative stress events and have been shown to prevent the degeneration of dopaminergic neurons in transgenic animal models [152]. With respect to neurodegenerative disease, the postmortem analysis of PD brains has revealed, in addition to *α*-syn and ubiquitin deposits, an increased concentration of iron [150]. Diverse iron distributions within the brain regions have been observed throughout the progression of PD. An explanation of this phenomenon can be found similarly in the differential expressions of iron trafficking and storage factors ferroportin and ferritin in the affected brain parts [151, 153]. Recent in vitro studies have shown that mutant *α*-syn interacts with metals and that iron (Fe^2+^, Fe^3+^) and copper (Cu^2+^) seem to aggravate the formation of thick *α*-syn fibrils and induce neuronal toxicity [154, 155]. This has been further confirmed in transgenic animal models and by the in vitro cellular overexpression of human *α*-syn, with a significant increase in the iron concentration and redistribution of iron from the cytoplasm to the perinuclear region within *α*-syn-rich inclusions [150]. On the other hand, aggregated *α*-syn provokes the metal ions (Fe^2+^, Mn^2+^) that mediate oxidative stress, thus closing the harmful circle between *α*-syn aggregation and the generation of oxidative stress [145, 150, 156, 157]. The chelated form of iron has also been identified in AD-specific A*β* plaques [83]. In the *Drosophila* model, iron chelated by A*β* plaques has been recognized as a primary location of intracellular free radical generation (Table 2), mainly by the Fenton reaction [158]. Rival and colleagues have even provided evidence that the expression of ferritin subunits and treatment with iron chelators are able to relieve A*β*-induced toxicity in the *Drosophila* model [159].

### 5.2. Copper and Zinc

Copper, as a metalloenzyme cofactor, is essential for normal brain development and function. Disregulation of its homeostasis has been implicated in PD, AD, HD, and ALS. In this context, free unbound copper is involved in oxidative stress and *α*-syn oligomerization and aggregation [160]. Copper ions display a decrease in their total concentration as reported in the *substantia nigra* in the majority of studies of PD patients [161]. In addition, copper regulates the iron levels in brain by ferroxidase ceruloplasmin activity. In PD patients, elevated levels of iron are accompanied by decreased levels of copper and ceruloplasmin in the brain; on the contrary, elevated levels of free copper result in decreased ferroxidase activity in the cerebrospinal fluid [160]. Copper is able to bind to *α*-syn and promotes its fibrillation and aggregation [162, 163]. In addition, the *α*-syn-copper complex alters the redox properties of copper and, thus, induces oxidative stress and even oxidizes several endogenous antioxidants, such as GSH [164]. DJ-1 (PARK7), the PD-linked redox-sensitive chaperone and oxidative stress sensor, inhibits *α*-syn aggregation and consequently neuronal cell loss [165]. This protein is also prone to bind copper and has a protective effect against copper-induced oxidative stress in cellular models [160]. This has further been supported by the evidence that mice lacking DJ-1 are more sensitive to MTPT exposure [166].

As has been well described, a higher intracellular concentration of ROS is also connected with the release of other biometals from their binding to metalloenzymes (Table 2). This is, in particular, valid for the Zn^2+^ cation whose elevated levels have been found mainly not only in the *substantia nigra* but also in other tissues of PD patients [161]. Recognizing the significant role of zinc, Ramirez and colleagues have demonstrated the higher vulnerability of human-originating neural cells to oxidative stress in cases of ATP13A2 (PARK9) deficiency. ATP13A2 (PARK9) belongs to the lysosomal type 5 P-type ATPase family [167]. Its cationic selectivity is still not determined, and the regulation of the homeostasis of several biometals, including Zn^2+^ and Mn^2+^, seems to be tightly bound to this ATPase. Furthermore, the chelation of Zn^2+^ ions by a specific Zn^2+^ chelator and the reintroduction of ATP13A2 into the deficient cells lead to a decrease in ROS-mediated toxicity [168].

In the context of AD, copper is another biometal (beside zinc and iron) that has been identified in the amyloid plaques of AD patients [162]. As early as 1999, White and colleagues showed that mice lacking amyloid precursor protein (APP) accumulated copper in the cortex and liver. On the contrary, mice overexpressing APP exhibited a decrease in the copper level in the brain [169]. Based on these data, the authors proposed that the amyloid precursor protein is a membrane-bound copper transporter [170]. Furthermore, both copper and zinc have the ability to bind to A*β* in vitro, and in neuronal cells, these interactions result in the generation of oxidative stress, A*β* aggregation, and neuronal cell loss. Interestingly, the affinity of copper to A*β* depends on the length of the A*β* species, with a higher affinity to A*β* (1–42) compared with A*β* (1–40). This also corresponds to the ability of A*β* (1–42) to reduce Cu^2+^ to Cu^3+^ and to its effect on the generation of oxidative stress and neurotoxicity [162].

### 5.3. Manganese

Manganese, an essential cofactor for enzymes including arginase, glutamine synthetase, and SOD2, is critical for normal development and biological functions [171, 172]. Various transporters and the binding of this metal to numerous proteins maintain the homeostatic level of manganese in cells [173, 174]. Excessive exposure to manganese is connected, in addition to manganism, with the development of neurodegenerative diseases [173, 174]. This condition is accompanied with typical neurodegenerative mechanisms including oxidative stress, the disruption of mitochondrial function followed by ATP depletion, protein aggregation, and the attenuation of neurotransmitter synthesis [109]. In this regard, manganese stress in dopaminergic neurons inhibits dopamine synthesis and induces dopamine release from intracellular stores [171]. In vitro studies of neuronal cells have provided evidence that excessive manganese exposure accelerates the expression of *α*-syn and promotes its fibrillation and aggregation [163]. On the contrary, *α*-syn overexpression in neuronal cellular models increases the sensitivity to manganese exposure [171, 175, 176].

Several studies have also reported the involvement of manganese in the regulation of leucine-rich repeat kinase 2- (LRRK2-) mediated pathogenesis in PD (Table 2). Mutation G2019S, with enhanced kinase activity, is linked with familiar PD [177]. In vitro studies have suggested that, in addition to Mg^2+^, Mn^2+^ may act as a cofactor of LRRK2 (PARK8) activity, with a preference for Mg^2+^. In contrast to wild-type LRRK2, G2019S-mutated LRRK2 shows equal catalytic rates in the presence of both Mg^2+^ and Mn^2+^ [171, 178, 179]. Based on these observations, LRRK2 has been suggested to act as a biological sensor of manganese levels, whereby wild-type LRRK2 reacts to increased manganese levels by a decrease in its kinase activity. On the other hand, PD-linked G2019S remains active, even under elevated levels of manganese, indicating the putative pathological mechanism [178].

Furthermore, at least in yeast and mammalian cells, ATP13A2 has been demonstrated to have protective effects against toxicity mediated by various metals, including Mn, Cd, Ni, and Se [180–182]. Mutations in ATP13A2 have been identified as the cause of Kufor-Rakeb syndrome, a juvenile recessive neurodegenerative disorder [167, 180]. As mentioned above, ATP13A2 is a lysosomal type 5 P-type ATPase [167], with a function in the regulation of biometal homeostasis. The protective role of ATP13A2 against *α*-syn-induced toxicity has also been confirmed in cellular and in animal models of PD [181].

In terms of AD, a manganese challenge in neuronal cell lines leads to the hyperphosphorylation of tau via extracellular signal-regulated kinase (ERK) mitogen-activated protein kinase (MAPK) activation. This is followed by glycogen synthase kinase-3*β* (GSK-3*β*) activation, which in general is associated with tau hyperphosphorylation and tau-mediated neuronal death [183].

### 5.4. Magnesium

Magnesium, the second most abundant intracellular cation, is critical for many biological processes including cellular energy, gene transcription, cellular growth, survival, and differentiation [184]. In the context of neurodegenerative diseases, magnesium is, in general, considered as a neuroprotective agent. Magnesium homeostasis and the role of the transient potential melastatin 7 (TRPM7) transporter have been implicated in neurodegenerative disease such as PD, AD, and HD [184]. Chronic low intake or deficiency in Mg^2+^ is considered as a high risk factor of PD development (Table 2) and dopaminergic neuron death and, in animal models, leads to a higher susceptibility to MPTP-mediated neurotoxicity [185–187]. On the other hand, supplementation with Mg^2+^ protects or decreases the risk of PD pathogenesis in animal models and in humans [188, 189]. Transgenic zebrafish carrying the mutated form of TRPM7 exhibit defects in dopamine generation or release, with a PD-like phenotype, which is, importantly, partially recovered by the administration of dopamine [184]. The specific mechanism of these outcomes cannot be described precisely with regard to the “crossroad” position of Mg^2+^ among the metabolic pathways or regulatory effects on various ion pumps, cotransporters, and hormone activities [190–192]. Indeed, the increase or stabilization of the intracellular concentration of Mg^2+^ presents one of the impacts of the antidepressant imipramine. In addition, according to one hypothesis, DJ-1 (PARK7) plays a key role in intracellular stabilization of Mg^2+^ by the androgen-receptor-mediated inhibition of the Mg^2+^ efflux effector SLC41A1 [76]. The stabilization or slight increase of intracellular Mg^2+^ concentration is, as mentioned above, connected with the proper or at least improved function of several ion pumps effective in ATP hydrolysis [88]. The mechanism of impact of DJ-1 on Mg^2+^ homeostasis can also be understood as representing one of the numerous connection points of oxidative status and ion balance regulation. DJ-1 is a protein deglycase, which can also act in a chaperone-like manner and regulate the aggregation state of *α*-syn. The ability of DJ-1 to protect cells against the formation of *α*-syn fibrils is dependent on the oxidative status. This molecular redox balance sensor has a minimal affinity to *α*-syn in the unoxidized state, but the oxidation of DJ-1 increases its affinity to *α*-syn and thus preserves it from toxic fibril formation [193].

## 6. Conclusions

### 6.1. Common Features of Metabolic Disorders and Neurodegenerative Diseases

The causative pathogenesis of both metabolic and neurodegenerative diseases involve common mechanisms. The symptomatology of neurodegenerative disorders is the result of pathophysiology occurring not only in nerve cells but also in other cell types. Similarly, the pathophysiology of metabolic disorders does not avoid neural tissues. Furthermore, extensive recently obtained data suggest a metabolic background to neurodegenerative diseases. Recent evidence has emerged increasingly supporting the hypothesis that AD, one of the most prevalent neurodegenerative diseases, should also be considered as a type 3 DM (T3DM).

The influence of ionic imbalances, in particular, the decrease and/or increase of biometal concentration, and the disturbances in their contents within the various types of peripheral cells or brain regions are considered to play an important role in the development of both types of diseases. The information summarized in this review refer to specific pathomechanisms that take into account the changes in the levels of biometals and their close relationship to inflammatory processes, alterations in energy metabolism, and the generation of oxidative stress. Intracellular reduction in energy supplementation caused by mitochondrial deficiency, hypoxic conditions, or inflammatory changes has been found to have relevant association with both peripheral and neuronal degenerative disorders. Oxidative stress, alternating between the causal factor and the consequence of the disease pathophysiology, should therefore be recognized as a hallmark of the majority of metabolic and neurodegenerative diseases.

## Figures and Tables

**Table 1:** Association of changes in reviewed biometal levels with the development of metabolism defects.

Biometal	Type of change +/−	Observed effects	Citation
Mg	+	↓ intracellular Ca^2+^ ↑ oxidative glucose breakdown by stimulation of PDH activity	[82, 83, 94] [82, 83]
	−	Positive correlation with obesity ↑ oxidative stress markers Hyperglycemia, insulin resistance ↑ proinflammatory cytokines ↓ function of ATP-dependent ion pumps	[74, 75] [76, 77] [59] [92, 93] [88, 191, 192, 194]
Zn	+	↓ glycemia	[103]
	−	↑ immune system reactivity ↓ lowered antioxidant capacity via downregulation of Nrf2	[99, 100] [36]
Cu/Mn	+	↑increased risk of T2DM poor glycemic control	[33, 102]
Se	−	Found in prediabetic patients together with lowered Zn^2+^ and Mg^2+^	[33]

PDH: pyruvate dehydrogenase; Nrf2: NF-E2-related factor 2.

**Table 2:** Role of reviewed biometals in neurodegenerative diseases.

Biometal	Type of change	Effect	Citation
Fe	Oxidized form (Fe^3+^)	(i) Promotes A*β* and *α*-syn aggregation (ii) Detected in AD and PD brains	[150, 158, 159, 195]
		↑ intracellular ROS generation	[145, 150, 156–158]
		↑ GSH oxidation	[150, 151]
Cu	Free/unbound	(i) Promotes aggregation of *α*-syn ↑ oxidative stress	[160, 162, 163]
	Decrease	↑ of Fe levels	[160]
		(i) Detected in substantia nigra of PD brains	[161]
Zn	Free/unbound	(i) Promotes aggregation of A*β*	[162]
	ATP13A2 deficiency	↑ intracellular free Zn^2+^ ↑ ROS production	[161, 167, 168]
Mn	Decrease	(i) LRRK2 G2019S (NOT wt) stays active under ↑ Mn → biological sensor of Mn levels	[171, 178, 179]
	ATP13A2 deficiency	↑ intracellular Mn^2+^ ↑ *α*-syn-induced toxicity	[167, 180, 181, 196]
	Increase	(i) Indirect hyperphosphorylation of tau (ii) Tau-mediated neuronal death	[183]
Mg	Decrease	(i) High risk factor of PD development (ii) Regulation of Mg homeostasis (iii) Protection against protein aggregation	[184–187]
		(iv) Dopamine generation defects	[184]
	Stabilization/slightly increase	(i) Protection against risk of PD development in animal models and humans	[77, 189]

LRRK2 G2019S: mutation type of leucine-rich repeat kinase 2; ATP13A2: probable cation-transporting ATPase 13A2; GSH: glutathione.
